# Does early intensive multifactorial therapy reduce modelled cardiovascular risk in individuals with screen-detected diabetes? Results from the ADDITION-Europe cluster randomized trial

**DOI:** 10.1111/dme.12410

**Published:** 2014-04-01

**Authors:** J A Black, S J Sharp, N J Wareham, A Sandbæk, G E H M Rutten, T Lauritzen, K Khunti, M J Davies, K Borch-Johnsen, S J Griffin, R K Simmons

**Affiliations:** 1 MRC Epidemiology Unit, Cambridge University Biomedical CampusCambridge, UK; 2 School of Public Health, Section of General Practice, University of AarhusAarhus, Denmark; 3 Julius Center for Health Sciences and Primary Care, University Medical Center UtrechtUtrecht, the Netherlands; 4 Diabetes Research Unit, Leicester Diabetes Centre, University of Leicester, Leicester General HospitalLeicester, UK; 5 Holbæk HospitalHolbæk, Denmark

## Abstract

**Aims:**

Little is known about the long-term effects of intensive multifactorial treatment early in the diabetes disease trajectory. In the absence of long-term data on hard outcomes, we described change in 10-year modelled cardiovascular risk in the 5 years following diagnosis, and quantified the impact of intensive treatment on 10-year modelled cardiovascular risk at 5 years.

**Methods:**

In a pragmatic, cluster-randomized, parallel-group trial in Denmark, the Netherlands and the UK, 3057 people with screen-detected Type 2 diabetes were randomized by general practice to receive (1) routine care of diabetes according to national guidelines (1379 patients) or (2) intensive multifactorial target-driven management (1678 patients). Ten-year modelled cardiovascular disease risk was calculated at baseline and 5 years using the UK Prospective Diabetes Study Risk Engine (version 3β).

**Results:**

Among 2101 individuals with complete data at follow up (73.4%), 10-year modelled cardiovascular disease risk was 27.3% (sd 13.9) at baseline and 21.3% (sd 13.8) at 5-year follow-up (intensive treatment group difference –6.9, sd 9.0; routine care group difference –5.0, sd 12.2). Modelled 10-year cardiovascular disease risk was lower in the intensive treatment group compared with the routine care group at 5 years, after adjustment for baseline cardiovascular disease risk and clustering (–2.0; 95% CI –3.1 to –0.9).

**Conclusions:**

Despite increasing age and diabetes duration, there was a decline in modelled cardiovascular disease risk in the 5 years following diagnosis. Compared with routine care, 10-year modelled cardiovascular disease risk was lower in the intensive treatment group at 5 years. Our results suggest that patients benefit from intensive treatment early in the diabetes disease trajectory, where the rate of cardiovascular disease risk progression may be slowed.

## Introduction

Type 2 diabetes is associated with significantly elevated all-cause and cardiovascular disease-related mortality, as well as a higher incidence of micro- and macrovascular disease. Among individuals with established diabetes, risk of cardiovascular disease and mortality can be reduced by intensive treatment of multiple risk factors, including blood pressure, cholesterol and glucose, although there remains some uncertainty about the merits of tight glycaemic control. Treatment of individual cardiovascular disease risk factors is also effective [1] but we know less about intensive treatment earlier in the disease trajectory. Long-term results from the UK Prospective Diabetes Study (UKPDS) suggest a beneficial effect of intensive treatment of glucose in those with shorter diabetes duration [2]. Promotion of opportunistic screening [3] and testing for diabetes in at-risk asymptomatic patients [4,5] will lead to a greater number of individuals being diagnosed early. However, there are a number of outstanding uncertainties that need to be resolved before intensive multifactorial treatment can be recommended in this patient group.

**What's new?**

- Little is known about intensive treatment of Type 2 diabetes early in the disease trajectory.
- In ADDITION-Europe, a cluster-randomized trial of multifactorial treatment vs. routine care among individuals with screen-detected diabetes, there was a decline in 10-year modelled cardiovascular disease risk in both trial groups in the 5 years following diagnosis.
- Compared with routine care, modest increases in intensity of treatment were associated with a small but significantly lower modelled cardiovascular disease risk value at 5 years.
- Practitioners should be encouraged to treat multiple risk factors intensively from diagnosis to reduce the cardiovascular burden of Type 2 diabetes.

ADDITION-Europe is a parallel-group randomized controlled trial exploring the effect of an intervention to promote intensive multifactorial treatment in a population with screen-detected Type 2 diabetes. Five-year results from the ADDITION-Europe trial show small but significant increases in treatment and reductions in many cardiovascular disease risk factors, but a non-significant 17% reduction in cardiovascular events [6]. Longer-term follow-up may be needed in order to establish whether early intensive treatment reduces cardiovascular risk [2].

In the absence of long-term data on hard outcomes, the difference in 10-year modelled cardiovascular disease risk at 5 years in ADDITION-Europe can shed light on the early cardiovascular disease experience of screen-detected individuals. We aimed to (1) describe the change in 10-year modelled cardiovascular risk in the 5 years following diagnosis with this screen-detected population and (2) quantify the impact of the intervention on 10-year modelled cardiovascular risk at 5 years.

## Methods

The design and rationale for the ADDITION-Europe trial have been previously reported (Clinical Trials Registry No; NCT 00237549) [6]. In brief, ADDITION-Europe is a primary-care-based study of a pragmatic cluster randomized controlled trial in a screen-detected diabetes population, comparing intensive multifactorial treatment with routine care in four centres (Cambridge, UK; Denmark; Leicester, UK; the Netherlands). Of 1312 general practices invited to participate, 379 (29%) agreed and 343 (26%) were independently randomized into routine care or intensive multifactorial treatment. Between April 2001 and December 2006, practices undertook stepwise screening of patients aged 40–69 years (50–69 years in the Netherlands), without known diabetes. Individuals were not invited for screening if they were pregnant or lactating, housebound, terminally ill with a prognosis of less than 12 months or had a psychiatric illness likely to invalidate consent. Individuals were diagnosed with diabetes according to World Health Organization (WHO) criteria [7]. Of the 3233 patients identified with diabetes by screening, 3057 (95%) consented to participate in the trial. The study was approved by local ethics committees in each centre. All participants provided written informed consent.

### Intervention

The characteristics of the interventions to promote intensive treatment in each centre have been described previously (http://www.addition.au.dk/) [6,8–11]. Family doctors, practice nurses and participants were educated in target-driven management (using medication and promotion of healthy lifestyles) of hyperglycaemia, blood pressure and cholesterol, based on the stepwise regimen used in the Steno-2 study [12]. The intervention delivered was practice based, except in Leicester, where patients also had access to individualized community clinics every 2 months [6,10]. Treatment targets and algorithms were based on trial data [6,8,13]. Targets included HbA_1c_ < 53 mmol/mol (7.0%) if HbA_1C_ > 47.5 mmol/mol (6.5%), blood pressure ≤ 135/85 mmHg if ≥ 120/80 mmHg, cholesterol < 5 mmol/l without ischaemic heart disease or < 4.5 mmol/l with ischaemic heart disease, and prescription of aspirin to those treated with anti-hypertensive medication. Statins were recommended to all patients with a cholesterol level ≥ 3.5 mmol/l following results from the Heart Protection Study [14]. Individuals in the routine care group received the standard pattern of diabetes care according to current recommendations in each centre.

### Measurement and outcomes

Trained staff independently assessed patients' health at baseline and after 5 years of follow-up by collecting biochemical and anthropometric data according to standard operating procedures. Self-report questionnaires were used to collect information on socio-demographic information, lifestyle habits and medication use. All staff collecting measurements were unaware of treatment group allocation. Changes in biochemical measures and medication from baseline to 5-year follow-up have been reported previously [6].

Individuals were followed for a mean of 5.7 years. The primary endpoint for this analysis was 10-year modelled cardiovascular disease risk, calculated from the UKPDS model (version 3β) [15], at 5 years post-diagnosis. This is a diabetes-specific risk assessment tool that estimates the absolute risk of fatal or non-fatal cardiovascular disease within a defined time frame up to 20 years. Participants with complete data on the UKPDS score variables at baseline and 5-year follow-up were assessed. The variables include age, gender, ethnicity, smoking status, HbA_1c_, systolic blood pressure, total-to-HDL cholesterol ratio, atrial fibrillation, previous myocardial infarction or stroke, microalbuminuria (albumin:creatine ratio ≥ 2.5 mg/mmol in men or ≥ 3.5 mg/mmol in women), macroalbuminuria (albumin:creatine ratio ≥ 30 mg/mmol), duration of diagnosed diabetes and BMI. We did not have data on atrial fibrillation in ADDITION-Europe participants, so all individuals were coded as zero (no atrial fibrillation). There was a large proportion of missing data for smoking at 5-year follow-up in the Netherlands (29%), so values from baseline were carried forward if missing at follow-up for all centres.

### Statistical analysis

Individuals who had died before 5-year follow up were excluded from all analyses. We summarized characteristics of ADDITION-Europe participants by trial group at baseline and 5-year follow-up. We report change from baseline to follow-up in each treatment group. Intermediate endpoints and modelled cardiovascular disease risk at 5 years were analysed within each centre using linear or logistic regression, with adjustment for the endpoint baseline values. A robust variance estimate based on practice level clustering was specified in the model. Centre-specific estimates of the difference between treatment groups were combined using fixed-effects meta-analysis. The *I*^2^ statistic was used to estimate heterogeneity between study centres [16].

In order to characterize missing data, we used logistic regression to model the odds of having a missing modelled risk score value at follow-up, adjusting for demographic and risk factor measurements as well as clustering at baseline. We also explored the impact of missing data at baseline and follow-up. First, individuals with missing modelled risk score at baseline were included in the analysis using the missing indicator method [17]. Then we extended the this analysis further with a pattern-mixture model [18], with the assumption that mean cardiovascular disease risk was, on average, 10% higher in individuals lost to follow-up.

We performed sensitivity analyses by (1) excluding individuals with prevalent or incident cardiovascular disease and (2) excluding those individuals with missing data for smoking at 5 years.

In all analyses, individuals were assigned to the groups to which they were originally randomized. Data were analysed using Stata version 12.1 (StataCorp., College Station, TX, USA).

## Results

### Participant characteristics

One hundred and ninety-six people were excluded as they died before 5-year follow-up (see Supporting Information, Fig. S1). A further 760 individuals were excluded as they did not have complete data to calculate the UKPDS risk score at baseline and follow-up, leaving 2101 (73%) participants with complete data for analysis. Participants who did not have data for modelled risk at follow-up were more likely to smoke at baseline (odds ratio 1.6; 95% CI 1.2–2.4) and be obese (BMI > 30 kg/m^2^, odds ratio 1.6; 95% CI 1.1–2.3) than those with complete data. No other differences between those lost to follow-up and the complete case analysis sample were found. Practices were well matched at baseline [6]. Overall, participants were well matched at baseline (Table 1). There were minor differences between groups at the centre level. Use of hypertensive and lipid-lowering drugs was higher in the intensive treatment group in Leicester. In Denmark, the intensive treatment group had a larger number of participants who reported previous myocardial infarction (6.2% vs. 4.5%) and stroke (2.6% vs. 1.3%) at baseline compared with the routine care group. Further, there were more patients with diabetes in the intensive treatment compared with the routine care group (837 and 579, respectively). Between centres, a lower prevalence of previous myocardial infarction or stroke at baseline was present in Denmark and in the Netherlands compared with the UK centres. All other values were similar between centres.

**Table 1:** Characteristics of the ADDITION-Europe trial cohort with complete data for the UK Prospective Diabetes Study Risk Engine (version 3β) at baseline and follow-up (mean 5.7 years)

	Routine care (*n* = 937)			Intensive treatment (*n* = 1164)			
			
Self reported	Baseline	Follow-up	Mean change from baseline to follow-up (sd)	Baseline	Follow-up	Mean change from baseline to follow-up (sd)	Intervention effect† β/odds ratio (95% CI)
Female sex	42%	—	—	41%	—	—	—
Mean (sd) age in years at diagnosis	59.9 (6.7)	—	—	60.1 (6.7)	—	—	—
White ethnicity	93%	—	—	96%	—	—	—
Employed	46%	—	—	42%	—	—	—
Any glucose-lowering drug	0.4%	57%	56%	0.6%	67%	67%	1.6 (1.3–2.0)
Any hypertensive drug	44%	74%	30%	46%	84%	37%	1.8 (1.3–2.3)
Any lipid-lowering drug	15%	78%	63%	18%	85%	67%	1.5 (1.1–1.9)
History of myocardial infarction	4.9%	—	—	7%	—	—	—
History of stroke	1.6%	—	—	2.6%	—	—	—
Current smoker	25%	20%	–4.6%	25%	20%	–4.9%	0.7 (0.4–1.1)
Median (p25, p75*) units of alcohol per week	5 (1–12)	4 (0–11)	–1.3 (8.7)	5 (1–13)	3 (0–10)	–1.3 (7.8)	–0.2 (–0.8 to 0.3)
Clinical							
Mean (sd) BMI in kg/m^2^	31.4 (5.4)	30.9 (5.5)	–0.5 (2.4)	31.6 (5.4)	31.1 (5.5)	–0.5 (2.6)	–0.03 (–0.2 to 0.2)
Median (p25, p75*) HbA_1c_ in mmol/mol and%	49 (43–56); 6.6 (6.1–7.3)	48 (43–54) 6.5 (6.1–7.1)	–3.3 (17.0); –0.3 (1.6)	48 (43–56); 6.5 (6.1–7.3)	46 (42–52); 6.4 (6.0–6.9)	–4.7 (15.8); –0.4 (1.4)	–0.9 (–1.7 to –0.1); –0.1 (–0.2 to –0.01)
Mean(sd) systolic blood pressure in mmHg	149.9 (21.4)	138.0 (17.6)	–11.8 (22.3)	148.1 (21.9)	135.0 (16.6)	–13.3 (21.9)	–3.0 (–4.9 to –1.1)
Mean (sd) total cholesterol:HDL in mmol/l	4.7 (1.5)	3.5 (1.0)	–1.2 (1.4)	4.7 (1.5)	3.3 (1.1)	–1.3 (1.4)	–0.1 (–0.2 to –0.06)
Mean (sd) LDL cholesterol in mmol/l	3.5 (1.0)	2.3 (0.8)	–1.2 (1.1)	3.4 (1.0)	2.0 (0.8)	–1.4 (1.1)	–0.2 (–0.3 to –0.1)
Median (p25, p75*) triglycerides in mmol/l	1.7 (1.2–2.4)	1.6 (1.1–2.3)	–0.3 (1.4)	1.6 (1.2–2.3)	1.5 (1.0–2.1)	–0.2 (1.3)	–0.04 (–0.1 to 0.03)
Median albumin:creatine ratio (p25, p75*)	0.86 (0.4–1.9)	1.1 (0.6–2.7)	1.7 (19.7)	0.8 (0.4–2.0)	1.2 (0.7–2.6)	1.5 (23.2)	–0.7 (–1.8 to 0.4)

* 25^th^ and 75^th^ percentile. All change values were normally distributed, so mean change and standard deviation (sd) are presented.

† Intervention effect is estimated from a meta-analysis of centre level linear or logistic regression model, with the characteristic at follow-up as the outcome, adjusted for baseline value and with robust standard errors allowing for clustering by general practice.

Prescription of cardio-protective drugs increased in both groups, with glucose-lowering, anti-hypertensive and lipid-lowering drugs more commonly prescribed in the intervention group at follow-up (Table 1). There were small but significant differences between groups for change in systolic blood pressure and total:HDL ratio and LDL cholesterol, in favour of the intensive treatment group (Table 1).

### Change in 10-year modelled cardiovascular disease risk

**FIGURE 1:**
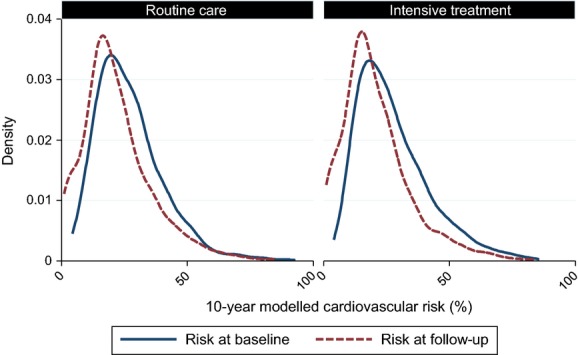
Distribution of 10-year modelled cardiovascular risk at baseline and 5.7-year follow-up in the ADDITION-Europe trial cohort by treatment group.

Ten-year modelled cardiovascular disease risk was 27.3% (sd 13.9) at baseline in the ADDITION-Europe trial cohort and 21.3% (sd 13.8) at 5 years (Table 2). Across all four centres there was a decline in modelled risk from baseline to follow-up in both the routine care (–5.0%; sd 12.2) and intensive care group (–6.9%; sd 9.0). Figure 1 shows the distribution of cardiovascular disease risk at baseline and follow-up separately by treatment group. For both groups, the distribution of modelled cardiovascular disease risk shifted to the left. Declines in modelled risk from diagnosis to 5 years were correlated with decreases in lipid, glucose and blood pressure values (see Supporting Information, Fig. S2).

**Table 2:** 10-year modelled cardiovascular disease risk (UK Prospective Diabetes Risk Engine version 3β) in the ADDITION-Europe trial cohort at baseline and 5.7 years by centre and combined

	Routine care				Intensive treatment			
		
Centre	Total with data* (% of randomized)	Mean at baseline (sd)	Mean at follow-up (sd)	Mean change baseline to follow-up (sd)	Total with data* (% of randomized)	Mean at baseline (sd)	Mean at follow-up (sd)	Mean change baseline to follow-up (sd)
Cambridge	285 (75%)	27.7 (13.8)	22.8 (14.1)	–5.1 (14.4)	334 (77%)	29.3 (15.1)	22.1 (14.4)	–6.4 (14.0)
Leicester	77 (81%)	23.9 (11.4)	19.9 (13.9)	–2.3 (9.5)	56 (93%)	27.5 (13.9)	19.0 (12.0)	–8.5 (11.2)
Denmark	423 (73%)	25.4 (12.2)	21.5 (13.7)	–3.7 (12.8)	594 (71%)	25.0 (13.2)	20.2 (13.7)	–4.7 (12.1)
The Netherlands	152 (66%)	33.6 (14.3)	23.3 (14.7)	–9.8 (16.3)	180 (73%)	35.8 (15.7)	20.7 (13.8)	–14.7 (11.4)
Combined	937 (73%)	27.4 (13.3)	22.1 (14.0)	–5.0 (12.2)	1164 (74%)	28.1 (14.7)	20.7 (13.8)	–6.9 (9.0)

* Total with risk score available at baseline and follow-up.

### Difference in 10-year modelled cardiovascular disease risk between groups at 5-year follow-up

**FIGURE 2:**
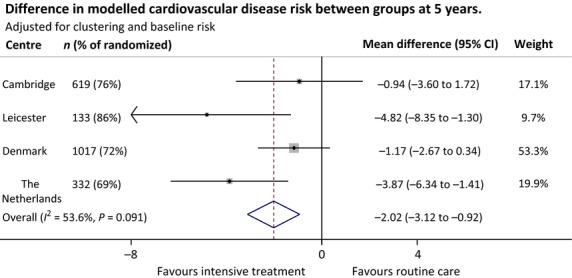
Difference in modelled cardiovascular disease risk between treatment groups at 5.7-year follow up in the ADDITION-Europe trial cohort, adjusted for baseline risk and accounting for clustering by general practice.

Within all four centres, cardiovascular disease risk was lower in the intensive treatment group compared with the routine care group at 5 years (Fig. 2). The difference between groups ranged from –0.9% (95% CI –3.6 to 1.7) in Cambridge to –4.8% (95% CI –8.4 to –1.3) in the Netherlands. There was moderate variation between centres (*I*^2^ = 53.6%). When results from each centre were combined, 10-year modelled cardiovascular disease risk was significantly lower: –2.0%; 95% CI –3.1 to –0.9 in the intensive treatment group, after adjustment for baseline cardiovascular disease risk and clustering. Sensitivity analyses suggest that this result was robust to data missing not at random (see Supporting Information, Fig. S3). Similarly, results remained the same when individuals with prevalent or incident cardiovascular disease were excluded, and when individuals with missing data for smoking at 5 years were excluded (see Supporting Information, Fig. S3).

## Discussion

In spite of increasing age and duration of diabetes, there was a decline in modelled cardiovascular disease risk in patients with diabetes in the 5 years following detection by screening. Further, compared with routine care, modest increases in intensity of treatment in the first 5 years after diagnosis were associated with improvements in cardiovascular disease risk factors, and with a small but significantly lower modelled cardiovascular disease risk value at 5 years (–2.0%; 95% CI –3.1 to –0.9). Our results highlight the importance for practitioners of intensively targeting cardiovascular risk factors early in the diabetes disease trajectory, where the rate of cardiovascular disease risk progression may be slowed.

### Comparison with other studies

A small but non-significant reduction in the relative hazard of the composite cardiovascular disease endpoint (hazard ratio 0.83; 95% CI 0.65–1.05) was present in the ADDITION-Europe trial at 5 years [6]. There are no other trial data from screen-detected diabetes populations with which to compare our results. However, similar improvements in the cardiovascular disease risk factors that drive modelled cardiovascular disease risk were seen in the patients with clinically diagnosed diabetes in the UKPDS trial at 6 years of follow-up [19]. Similar decreases in cardiovascular disease risk factor values in the 12 months following diagnosis have been reported among newly diagnosed patients enrolled in cardiovascular disease risk reduction lifestyle interventions [20,21]. While there is a lack of studies intervening early in the diabetes disease trajectory, our results are supported by studies of individuals with established diabetes, for example, in the multifactorial Steno-2 study[12,21], as well as similar 1-year modelled risk improvements in two trials of pharmacist-led behavioural advice compared to routine care [23,24].

In ADDITION-Europe, 5.3% of individuals in the routine care group experienced a myocardial infarction or stroke in the first 5 years, compared with 9.3% of the routine care group in the first 6 years of follow-up in the younger UKPDS cohort (mean age 53 vs. 60 years) [19]. While the length of follow-up differs, it is likely that the extent of the difference is attributable to underlying changes in routine care. At baseline in the UKPDS, which began recruitment two decades before ADDITION-Europe, 12% of patients were prescribed blood pressure-lowering medication and 0.3% of individuals were prescribed lipid-lowering medication [13]. In ADDITION-Europe, at baseline, 45% were prescribed anti-hypertensive medication and 16% were prescribed lipid-lowering medication. This suggests that cardiovascular disease prevention in populations at risk of diabetes has improved between the recruitment phases of the two studies. Furthermore, the delivery of diabetes care in the general practice setting continued to improve throughout the trial. The introduction of the Quality and Outcomes Framework in the UK and evidence-based guidelines in the Netherlands and Denmark, as well as general promotion of cardiovascular disease risk management in people with diabetes [25–27], may have decreased the potential to achieve a difference in treatment and thus a larger difference in cardiovascular disease risk between groups [25].

### Strengths and limitations

ADDITION-Europe participants were recruited from a large population-based sample in three European countries. Participants were diagnosed according to WHO criteria. Randomizing general practices reduced the risk of intervention contamination. Treatment guidelines across the centres at baseline were similar [25–28], but centres were encouraged to implement screening and treatment algorithms to suit their local environment. Participant retention was high at follow-up. We assessed clinically important outcomes using standard operating procedures and staff were blind to treatment allocation. Overall, 27% of data were missing from the primary analysis. The effect of missing data at baseline and follow-up was explored using methods appropriate for a trial, and results suggested that the primary analysis likely represented an accurate intent-to-treat analysis. Derived from over 40 000 patient-years of data and 1115 cardiovascular disease events [15], the latest refinement of the UKPDS risk score is the most appropriate tool for predicting 10-year modelled cardiovascular disease risk in this population [29]. Modelled cardiovascular risk may have been overestimated in our contemporary cohort as routine care after diagnosis is more intensive than that experienced by the UKPDS population. This would not have altered our effect size estimates differentially by group. Clinically diagnosed atrial fibrillation was unavailable, and this variable was set to 0 in the UKPDS model. As there was no difference in self-reported atrial fibrillation at 5 years between routine care (13.3%) and intensive treatment groups (14.0%), it is unlikely that inclusion of this variable in the UKPDS model would affect our main findings.

People that died between baseline and follow-up were excluded from this analysis (*n* = 196). While 24% (*n* = 48) of these deaths were attributed to cardiovascular disease, 1.6% (22/1377) of the routine care group and 1.5% (26/1678) of the intensive treatment group experienced a cardiovascular disease-related death. By excluding the 196 incident deaths before follow-up, it is likely that we have slightly underestimated the effect of intensive treatment on modelled cardiovascular disease risk. Participants were predominantly of white ethnic origin (93%), potentially limiting the extrapolation of these findings to more ethnically diverse centres. However, as prevention of diabetes-related complications in ethnic minorities is also effective [30], it is likely that the finding in favour of the intervention would remain. The most notable difference in the application of the treatment algorithm was in Leicester, where the education components of the intervention were delivered through the DESMOND structured education programme (http://www.desmond-project.org.uk/). Further differences were seen in Denmark, where practices completed opportunistic screening, potentially leading to over-selection of those at increased risk at baseline. It is likely these influences, in combination with differences in national characteristics across centres, accounted for most of the 54% of heterogeneity not attributable to chance identified in the analysis (*I*^2^ statistic 53.6%).

### Implications for practice

Previous literature has indicated that the benefits of intensive treatment are not restricted to those at highest risk [31]. After receiving the diagnostic label of diabetes, many ADDITION-Europe participants were prescribed treatment for multiple cardiovascular disease risk factors [6] and there was a decline in modelled cardiovascular disease risk across the whole risk distribution from baseline to 5-year follow-up. This has important implications for diabetes treatment. The American Diabetes Association recommends that diabetes testing should be considered in adults of any age with a BMI ≥ 25 kg/m^2^ and one or more known risk factors for diabetes [5]. Screening guidelines or programmes have also been introduced in the UK [4], Canada [32] and Australia [33]. These recommendations are likely to result in an increased number of individuals detected earlier in the disease trajectory. If early detection followed by intensive treatment, or even followed by the high standard of routine care now offered by primary care providers, leads to a population level shift in cardiovascular disease risk, it is likely that a large number of cardiovascular disease events might be averted. Small increases in treatment were not associated with a significant reduction in risk of events within 5 years [6], but were associated with a significant reduction in modelled events from 5 to 15 years. This suggests long-term follow-up of ADDITION-Europe beyond 5 years may mirror post-trial findings from the UKPDS study [2]. Future research should examine (1) whether this slowing of cardiovascular disease risk progression in the first 5 years after diagnosis leads to a sustained reduction in actual cardiovascular disease events over a longer follow-up time and (2) which individuals achieved more risk reduction than others to inform the development and targeting of future interventions.

## Conclusion

When compared with routine care, a modest increase in the treatment of risk factors among patients with Type 2 diabetes in the first 5 years after detection by screening was associated with a small but significant reduction in 10-year modelled cardiovascular disease risk at 5 years. Furthermore, cardiovascular disease risk estimates declined across the whole cohort from baseline to follow-up, in spite of increases in age and diabetes duration. Health practitioners are therefore encouraged to treat multiple cardiovascular risk factors early and intensively in the diabetes disease trajectory, where the rate of cardiovascular disease risk progression may be slowed. Longer-term follow-up of outcomes in the ADDITION-Europe trial cohort, alongside examination of microvascular, quality of life and cost data, is planned to establish the cost-effectiveness of early intensive treatment among screen-detected patients.

### Funding sources

ADDITION-Cambridge was supported by the Wellcome Trust (grant reference no. G061895); the Medical Research Council (grant reference no. G0001164); the National Institute for Health Research (NIHR) Health Technology Assessment Programme (grant reference no. 08/116/300); National Health Service R&D support funding (including the Primary Care Research and Diabetes Research Networks); and the National Institute for Health Research. SJG received support from the Department of Health NIHR Programme Grant funding scheme (RP-PG-0606-1259). The views expressed in this publication are those of the authors and not necessarily those of the UK Department of Health. Bio-Rad provided equipment for HbA_1c_ testing during the screening phase. ADDITION-Denmark was supported by the National Health Services in the counties of Copenhagen, Aarhus, Ringkøbing, Ribe and South Jutland in Denmark; the Danish Council for Strategic Research; the Danish Research Foundation for General Practice; Novo Nordisk Foundation; the Danish Centre for Evaluation and Health Technology Assessment; the diabetes fund of the National Board of Health; the Danish Medical Research Council; and the Aarhus University Research Foundation. The trial has been given unrestricted grants from Novo Nordisk AS, Novo Nordisk Scandinavia AB, Novo Nordisk UK, ASTRA Denmark, Pfizer Denmark, GlaxoSmithKline Pharma Denmark, Servier Denmark A/S and HemoCue Denmark A/S. Parts of the grants from Novo Nordisk Foundation, Danish Council for Strategic Research and Novo Nordisk were transferred to the other centres. ADDITION-Leicester was supported by Department of Health and ad hoc Support Sciences; the NIHR Health Technology Assessment Programme (grant reference no. 08/116/300); National Health Service R&D support funding (including the Primary Care Research and Diabetes Research Network, and LNR CLAHRC); and the National Institute for Health Research. MJD and KK receive support from the Department of Health NIHR Programme Grant funding scheme (RP-PG-0606-1272). ADDITION-Netherlands was supported by unrestricted grants from Novo Nordisk, Glaxo Smith Kline and Merck; and by the Julius Center for Health Sciences and Primary Care, University Medical Center, Utrecht.

The sponsors did not participate in the design or conduct of this study; in the collection, management, analysis, or interpretation of data; in the writing of the manuscript; or in the preparation, review, approval, or decision to submit this manuscript for publication.

### Competing interests

SJG and NJW have received an honorarium and travel expenses from Eli Lilly associated with membership of a data monitoring committee, and payment for preparing and delivering educational material from Novo Nordisk and the NHS; KB-J was director of the Steno Diabetes Centre, which is owned by Novo Nordisk, and holds stock in Novo Nordisk; MJD has served on advisory boards for Novo Nordisk, Eli Lilly, MSD, Bristol-Myers Squibb and Roche, and has received honoraria for speaking from Novo Nordisk, Eli Lilly, Sanofi-Aventis, Novartis and MSD; KK has participated in advisory boards for Novo Nordisk, Eli Lilly, MSD, Boehringer Ingelheim and Roche, and has received honoraria for speaking from Novo Nordisk, Eli Lilly, Sanofi-Aventis, Novartis and MSD; GEHMR has served as a consultant and participated in advisory boards for Novo Nordisk and MSD, and has received honoraria for speaking from Novo Nordisk; SJS, AS, JAB and RKS declare that they have no competing interests.

## List of Investigators Involved in All Centres

### ADDITION-Denmark

Bendix Carstensen, Merete Frandsen (Steno Diabetes Centre, Gentofte); Else-Marie Dalsgaard, Ynna Nielsen, Soren Bech-Morsing, Mette Vinther Skriver, Helle Terkildsen, Morten Charles (School of Public Health, Aarhus); Toke Bek (Aarhus University Hospital, Aarhus); Henrik Lund Andersen (Glostrup Hospital, Glostrup).

### ADDITION-Cambridge

Gisela Baker, Daniel Barnes, Mark Betts, Clare Boothby, Sandra Bovan, Parinya Chamnan, Sue Emms, Francis Finucane, Susie Hennings, Muriel Hood, Garry King, Christine May Hall, Joanna Mitchell, Emanuella De Lucia Rolfe, Liz White (MRC Epidemiology Unit, Cambridge); Amanda Adler, Sean Dinneen, Mark Evans (Cambridge University Hospitals, NHS Foundation Trust, Cambridge); Judith Argles, Rebecca Bale, Roslyn Barling, Sue Boase, Ryan Butler, Pesheya Doubleday, Tom Fanshawe, Philippa Gash, Julie Grant, Wendy Hardeman, Ann-Louise Kinmonth, Richard Parker, Nicola Popplewell, A Toby Prevost, Megan Smith, Stephen Sutton, Fiona Whittle, Kate Williams, Rebecca Abbott, Georgina Lewis, Lincoln Sargeant (Department of Public Health and Primary Care, University of Cambridge); Robert Henderson (Hinchingbrooke Hospital, Huntingdon).

### ADDITION-Netherlands

Kees Gorter, Paul Janssen, Lidian Izeboud, Jacqueline Berends, Marlies Blijleven, Bart Thoolen, Denise de Ridder, Jozien Bensing, Mehmet Akarsubasi, Paula Koekkoek, Carla Ruis, Geert Jan Biessels, Jaap Kappelle, Michiel van der Linden (Utrecht University, Utrecht).

### ADDITION-Leicester

Balasubramanian Thiagarajan Srinivasan, David Webb, Mary Quinn, Emma Wilmot, Samiul A Mostafa, Nitin Gholap, Hamid Mani, Winston Crasto, Steve Hiles, Joe Henson, Janet Jarvis, Sukhjit Sehmi, Fiona Ablett, Champa Merry, Emma Healey, Julia Stockman, Sandra Campbell, Janette Barnett, Nil Radia, Mo Radia, Jo Howe, Lesley Bryan, Jane Brela, Jayne Hill, Helen Bray, Rachel Plummer, Zubeir Essat, Francis Pullen, Susan Enright (University Hospitals of Leicester NHS Trust); Laura J Gray, Nick Taub (University of Leicester). University Medical Centre, Utrecht, the Netherlands).

## Supporting Information

**Figure S1.** CONSORT diagram of the ADDITION-Europe trial.

**Figure S2.** Correlation between change values of clinical risk factors and modelled risk from diagnosis to 5 years in ADDITION-Europe.

**Figure S3.** Sensitivity analysis of smoking and cardiovascular disease assumptions and effect of missing data at baseline and follow-up on the difference in the UKPDS Risk Engine (version 3β) modelled cardiovascular disease risk score between treatment groups at 5.7-year follow-up in the ADDITION-Europe trial cohort.
