# Supplementary material for: Does early intensive multifactorial therapy reduce modelled cardiovascular risk in individuals with screen-detected diabetes? Results from the ADDITION-Europe cluster randomized trial
Source: Diabet Med. 2014 Apr 1;31(6):647–56. doi: 10.1111/dme.12410 (PMC4150529; doi:10.1111/dme.12410)
Supplement: Supplementary file 1 — Figure S1. CONSORT diagram of the ADDITION-Europe trial. [file dme0031-0647-SD1.pptx]

## Slide 1
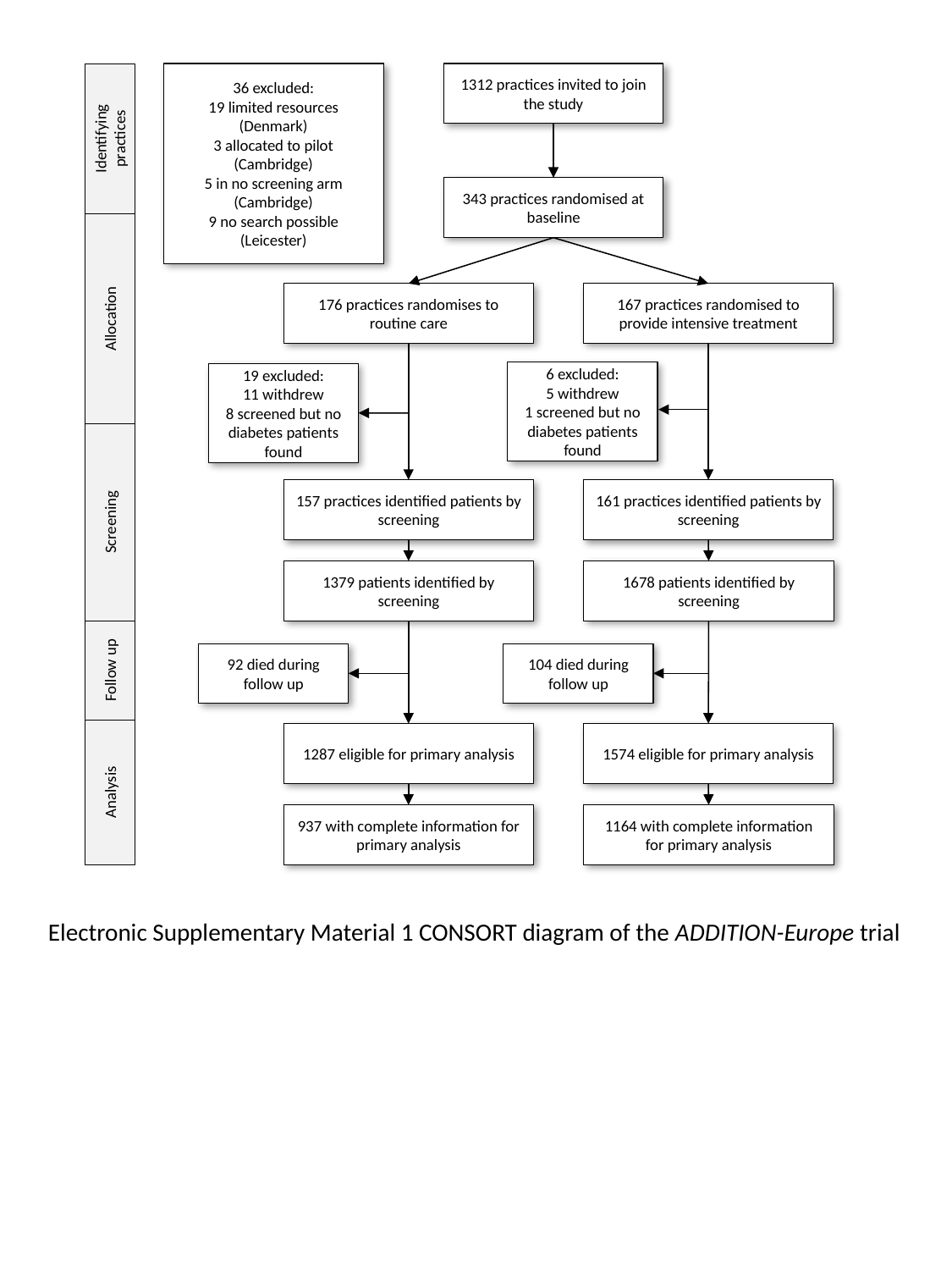

Identifying practices
36 excluded:
19 limited resources (Denmark)
3 allocated to pilot
(Cambridge)
5 in no screening arm
(Cambridge)
9 no search possible
(Leicester)
1312 practices invited to join the study
343 practices randomised at baseline
Allocation
176 practices randomises to routine care
167 practices randomised to provide intensive treatment
6 excluded:
5 withdrew
1 screened but no diabetes patients found
19 excluded:
11 withdrew
8 screened but no diabetes patients found
Screening
157 practices identified patients by screening
161 practices identified patients by screening
1379 patients identified by screening
1678 patients identified by screening
Follow up
92 died during follow up
104 died during follow up
Analysis
1287 eligible for primary analysis
1574 eligible for primary analysis
937 with complete information for primary analysis
1164 with complete information for primary analysis
Electronic Supplementary Material 1 CONSORT diagram of the ADDITION-Europe trial
