# Supplementary material for: Does early intensive multifactorial therapy reduce modelled cardiovascular risk in individuals with screen-detected diabetes? Results from the ADDITION-Europe cluster randomized trial
Source: Diabet Med. 2014 Apr 1;31(6):647–56. doi: 10.1111/dme.12410 (PMC4150529; doi:10.1111/dme.12410)
Supplement: Supplementary file 2 — Figure S2. Correlation between change values of clinical risk factors and modelled risk from diagnosis to 5 years in ADDITION-Europe. [file dme0031-0647-SD2.pptx]

## Slide 1
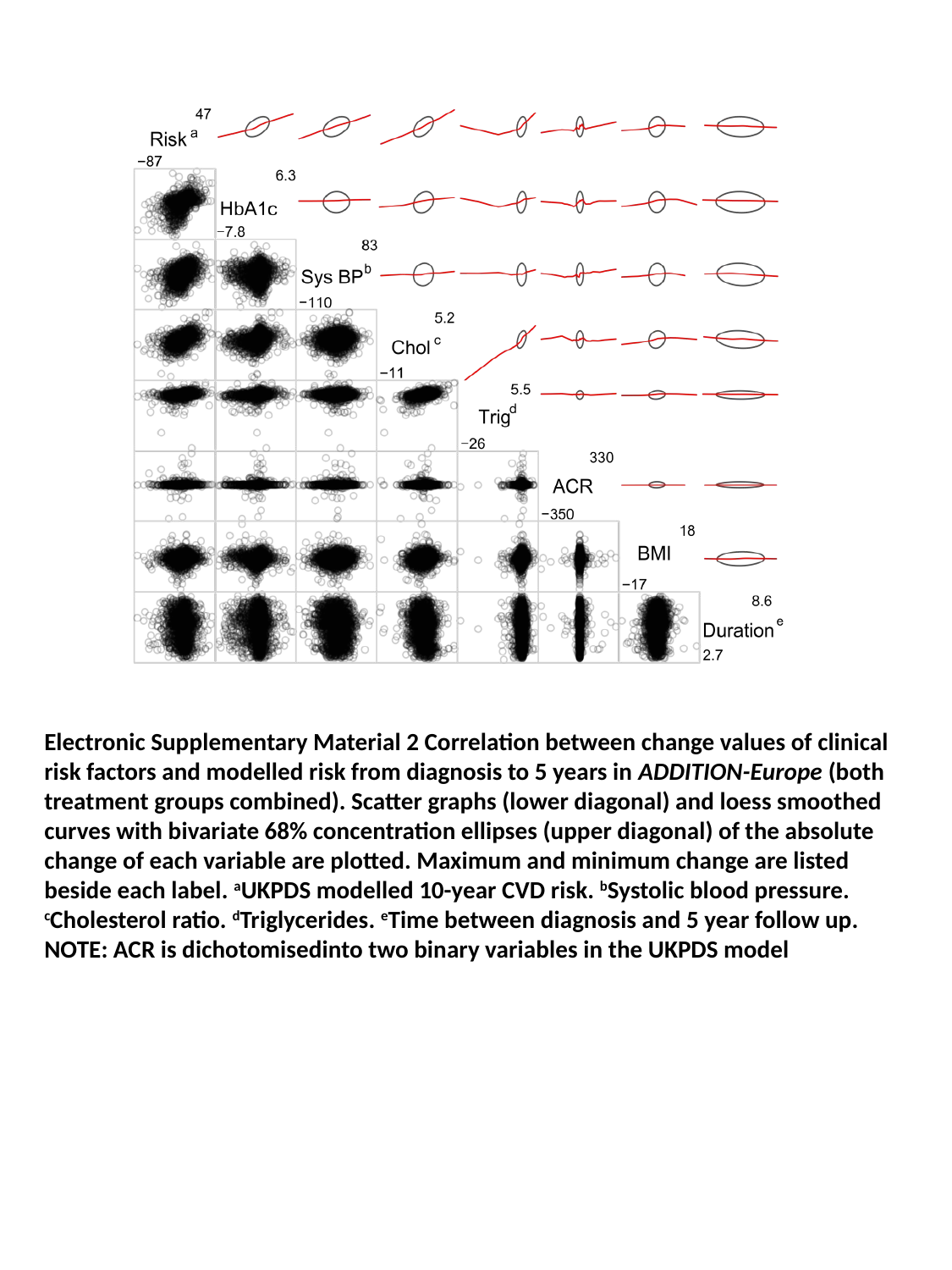

Electronic Supplementary Material 2 Correlation between change values of clinical risk factors and modelled risk from diagnosis to 5 years in ADDITION-Europe (both treatment groups combined). Scatter graphs (lower diagonal) and loess smoothed curves with bivariate 68% concentration ellipses (upper diagonal) of the absolute change of each variable are plotted. Maximum and minimum change are listed beside each label. aUKPDS modelled 10-year CVD risk. bSystolic blood pressure. cCholesterol ratio. dTriglycerides. eTime between diagnosis and 5 year follow up. NOTE: ACR is dichotomisedinto two binary variables in the UKPDS model
