# Supplementary material for: Does early intensive multifactorial therapy reduce modelled cardiovascular risk in individuals with screen-detected diabetes? Results from the ADDITION-Europe cluster randomized trial
Source: Diabet Med. 2014 Apr 1;31(6):647–56. doi: 10.1111/dme.12410 (PMC4150529; doi:10.1111/dme.12410)
Supplement: Supplementary file 3 — Figure S3. Sensitivity analysis of smoking and cardiovascular disease assumptions and effect of missing data at baseline and follow-up on the difference in the UKPDS Risk Engine (version 3β) modelled cardiovascular disease risk score between treatment groups at 5.7-year follow-up in the ADDITION-Europe trial cohort. [file dme0031-0647-SD3.pptx]

## Slide 1
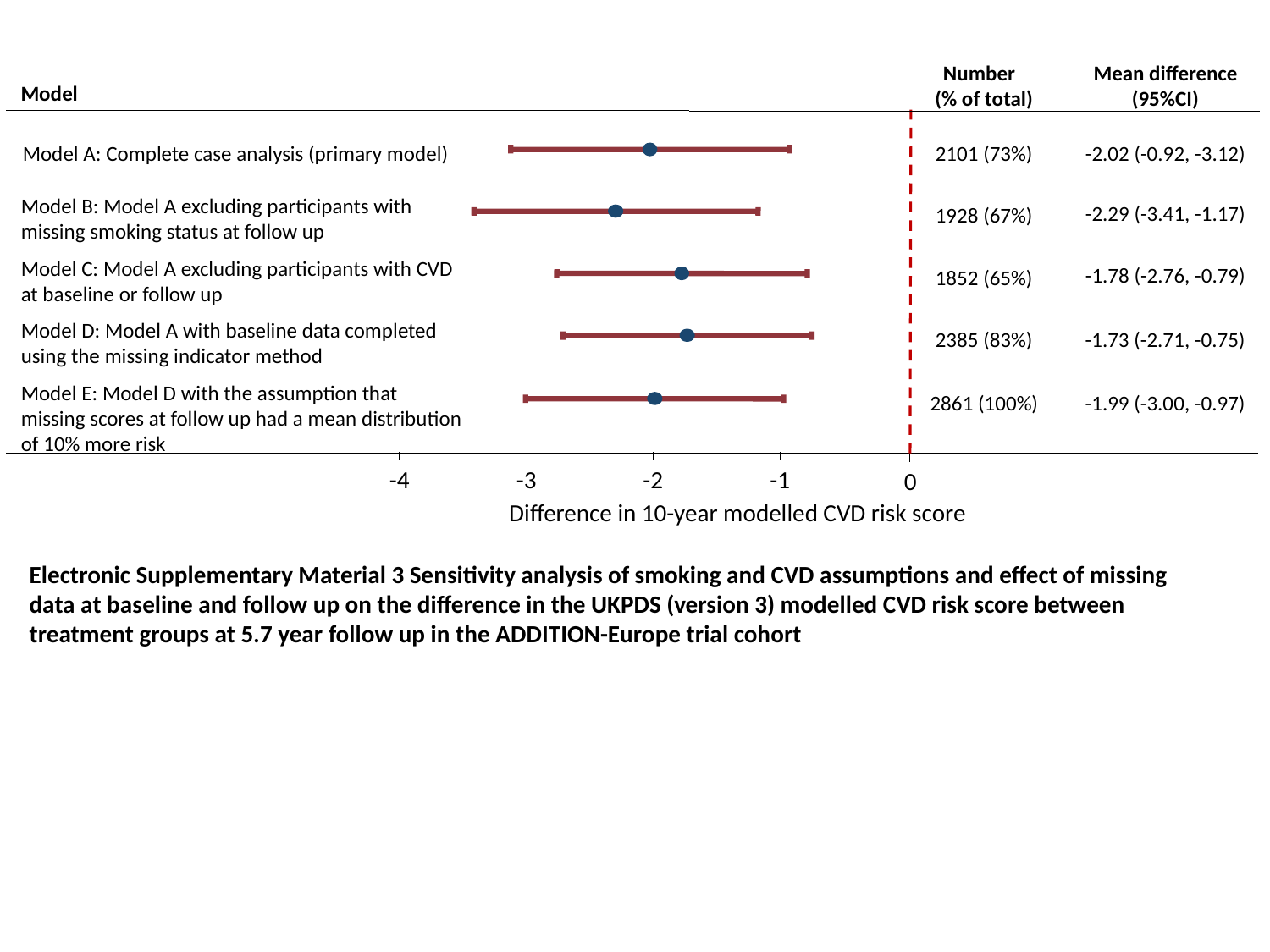

Number
(% of total)
Mean difference
(95%CI)
Model
Model A: Complete case analysis (primary model)
2101 (73%)
-2.02 (-0.92, -3.12)
Model B: Model A excluding participants with missing smoking status at follow up
-2.29 (-3.41, -1.17)
1928 (67%)
Model C: Model A excluding participants with CVD at baseline or follow up
-1.78 (-2.76, -0.79)
1852 (65%)
Model D: Model A with baseline data completed using the missing indicator method
2385 (83%)
-1.73 (-2.71, -0.75)
Model E: Model D with the assumption that missing scores at follow up had a mean distribution of 10% more risk
2861 (100%)
-1.99 (-3.00, -0.97)
-4
-3
-2
-1
0
Difference in 10-year modelled CVD risk score
Electronic Supplementary Material 3 Sensitivity analysis of smoking and CVD assumptions and effect of missing data at baseline and follow up on the difference in the UKPDS (version 3) modelled CVD risk score between treatment groups at 5.7 year follow up in the ADDITION-Europe trial cohort
