# Supplementary material for: Does early intensive multifactorial therapy reduce modelled cardiovascular risk in individuals with screen-detected diabetes? Results from the ADDITION-Europe cluster randomized trial
Source: Diabet Med. 2014 Apr 1;31(6):647–56. doi: 10.1111/dme.12410 (PMC4150529; doi:10.1111/dme.12410)
Supplement: Supplementary file 4 — Figure S3. Sensitivity analysis of smoking and cardiovascular disease assumptions and effect of missing data at baseline and follow-up on the difference in the UKPDS Risk Engine (version 3β) modelled cardiovascular disease risk score between treatment groups at 5.7-year follow-up in the ADDITION-Europe trial cohort. [file dme0031-0647-SD4.pptx]

## Slide 1
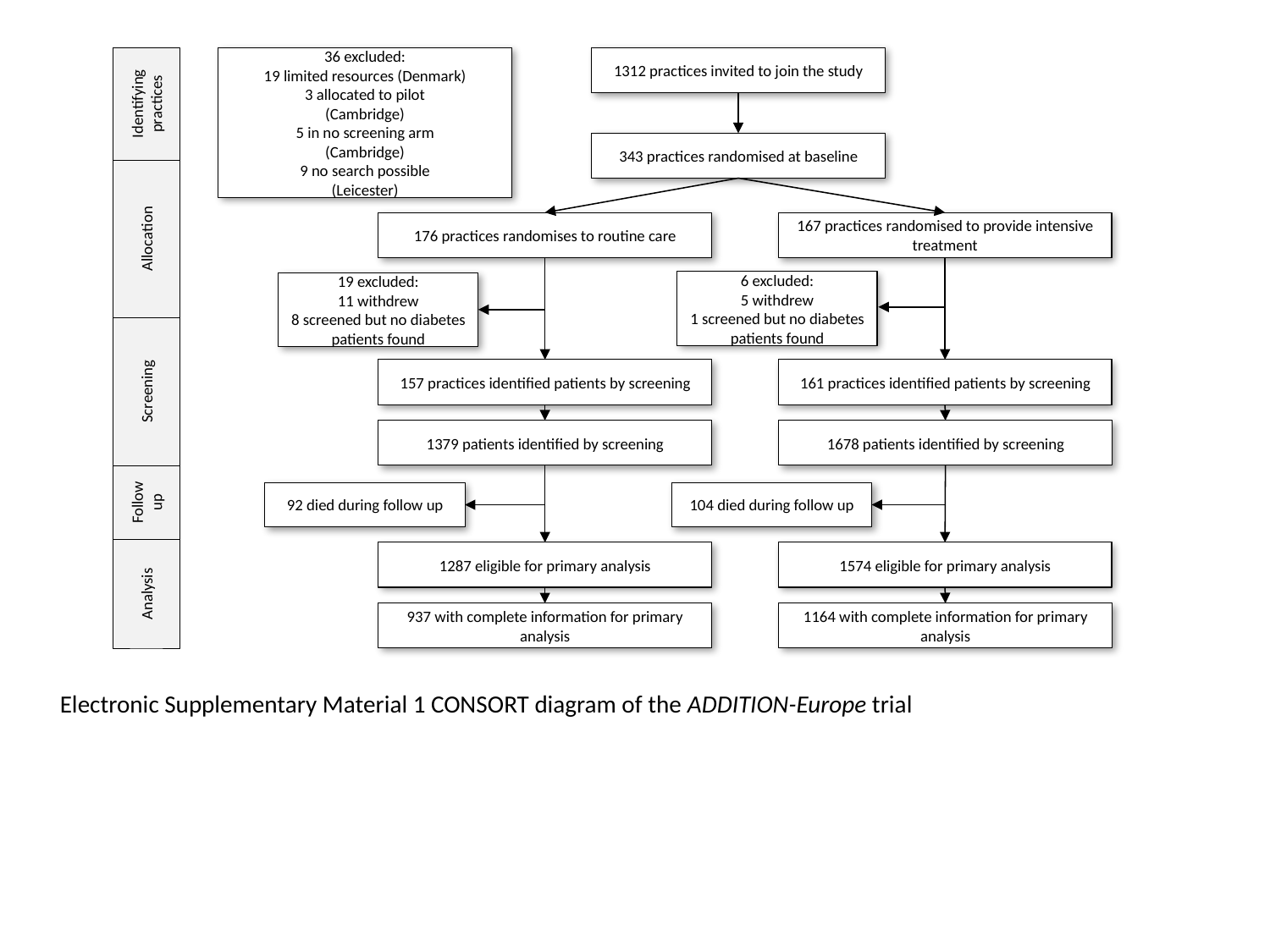

Identifying practices
36 excluded:
19 limited resources (Denmark)
3 allocated to pilot
(Cambridge)
5 in no screening arm
(Cambridge)
9 no search possible
(Leicester)
1312 practices invited to join the study
343 practices randomised at baseline
Allocation
176 practices randomises to routine care
167 practices randomised to provide intensive treatment
6 excluded:
5 withdrew
1 screened but no diabetes patients found
19 excluded:
11 withdrew
8 screened but no diabetes patients found
Screening
157 practices identified patients by screening
161 practices identified patients by screening
1379 patients identified by screening
1678 patients identified by screening
Follow up
92 died during follow up
104 died during follow up
Analysis
1287 eligible for primary analysis
1574 eligible for primary analysis
937 with complete information for primary analysis
1164 with complete information for primary analysis
Electronic Supplementary Material 1 CONSORT diagram of the ADDITION-Europe trial

## Slide 2
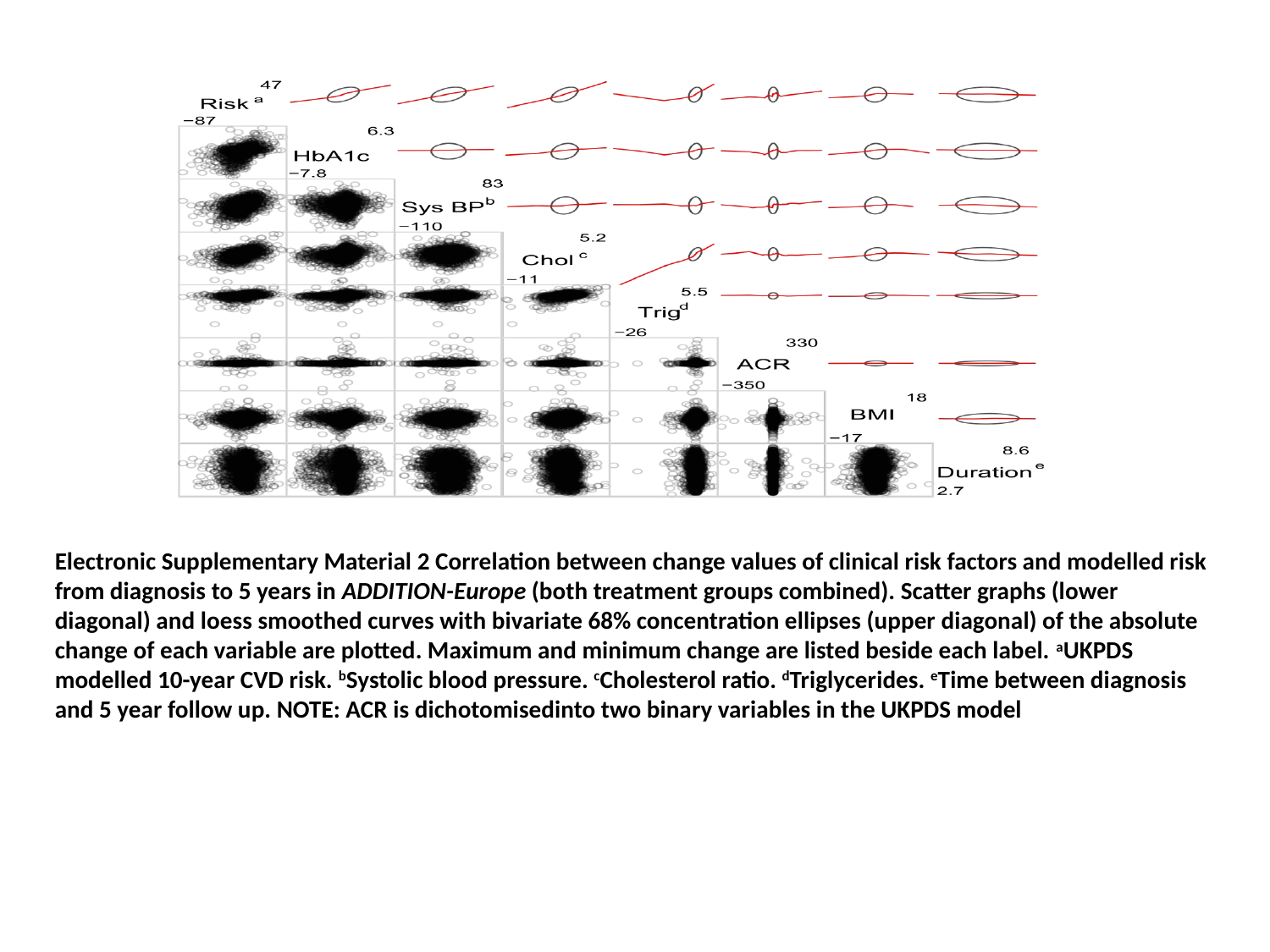

Electronic Supplementary Material 2 Correlation between change values of clinical risk factors and modelled risk from diagnosis to 5 years in ADDITION-Europe (both treatment groups combined). Scatter graphs (lower diagonal) and loess smoothed curves with bivariate 68% concentration ellipses (upper diagonal) of the absolute change of each variable are plotted. Maximum and minimum change are listed beside each label. aUKPDS modelled 10-year CVD risk. bSystolic blood pressure. cCholesterol ratio. dTriglycerides. eTime between diagnosis and 5 year follow up. NOTE: ACR is dichotomisedinto two binary variables in the UKPDS model

## Slide 3
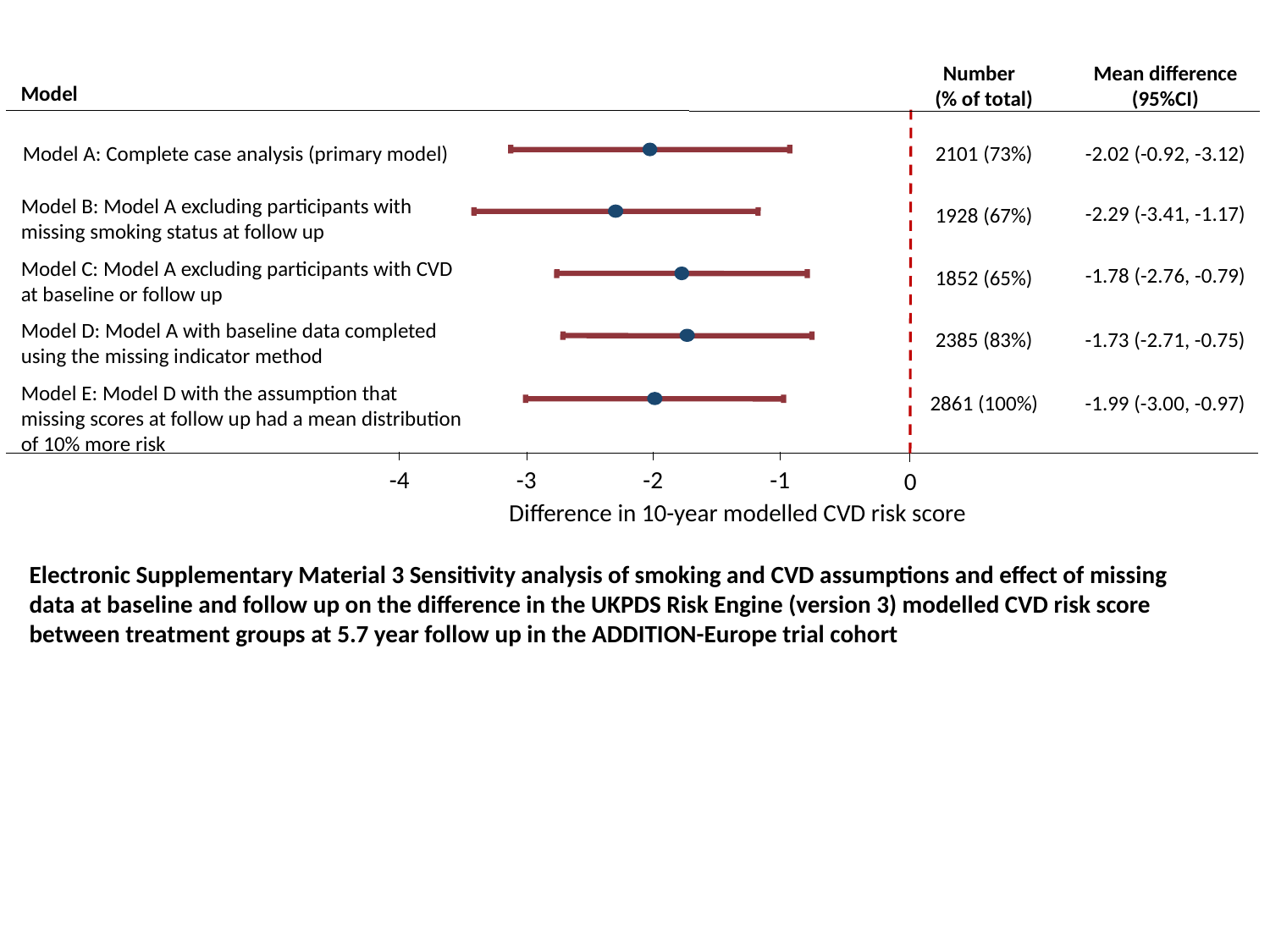

Number
(% of total)
Mean difference
(95%CI)
Model
Model A: Complete case analysis (primary model)
2101 (73%)
-2.02 (-0.92, -3.12)
Model B: Model A excluding participants with missing smoking status at follow up
-2.29 (-3.41, -1.17)
1928 (67%)
Model C: Model A excluding participants with CVD at baseline or follow up
-1.78 (-2.76, -0.79)
1852 (65%)
Model D: Model A with baseline data completed using the missing indicator method
2385 (83%)
-1.73 (-2.71, -0.75)
Model E: Model D with the assumption that missing scores at follow up had a mean distribution of 10% more risk
2861 (100%)
-1.99 (-3.00, -0.97)
-4
-3
-2
-1
0
Difference in 10-year modelled CVD risk score
Electronic Supplementary Material 3 Sensitivity analysis of smoking and CVD assumptions and effect of missing data at baseline and follow up on the difference in the UKPDS Risk Engine (version 3) modelled CVD risk score between treatment groups at 5.7 year follow up in the ADDITION-Europe trial cohort
